# Modelling the Potential Distribution of African Wormwood (*Artemisia afra*) Using a Machine Learning Algorithm‐Based Approach (MaxEnt) in Sekhukhune District, South Africa

**DOI:** 10.1002/ece3.71866

**Published:** 2025-08-18

**Authors:** Willem Matsane, Inos Dhau, Mologadi Clodean Mothapo, Kgabo Humphrey Thamaga

**Affiliations:** ^1^ Department of Geography and Environmental Studies University of Limpopo Sovenga South Africa; ^2^ Department of GIS and Remote Sensing University of Fort Hare Alice South Africa

**Keywords:** *Artemisia afra*, bioclimatic data, environmental variables, MaxEnt, receiver operating characteristic curve, species distribution models, suitable habitat prediction

## Abstract

*Artemisia afra* Jacq. ex Willd, commonly known as African wormwood, is a native medicinal plant that has been unsustainably harvested primarily for its leaves because of their medicinal properties. The unsustainable harvesting of this plant underscores the urgent need for conservation and management practices. This study, therefore, used the MaxEnt model of the potential distribution of *A. afra.* Sekhukhune District Municipality, South Africa. We used 105 sampled records and 27 environmental variables to model the potential spatial distribution of *A. afra* using the MaxEnt modeling approach. The predictions were performed using current climatic and topographic conditions. A significant portion of the area, 54.46%, is highly suitable for the distribution of *A. afra* , with various degrees. Precipitation contributed 33.6% to the suitability predictions, followed by NDVI, soil, and distance from rivers with 27.1%, 8.1%, and 5.7% respectively. *Artemisia afra* is predicted to be persistent in mountainous areas and along riverbanks. Higher elevated areas from 1000 to 1700 m are highly suitable for the persistence of *A. afra* species, as it remains cool and relatively moist under the changing climate. Conservation efforts should be focused on mountainous areas and along riverbanks. Rivers such as the Ngwaritsi, Motsephiri, and Steelpoort are in areas with highly suitable predictions. On the basis of the findings, we recommend conservation and management of highly suitable areas. A stakeholder‐inclusive conservation framework is proposed to guide community‐based protection of *A. afra* habitats.

## Graphical abstract

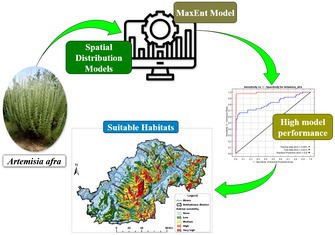

## 1 Introduction

African wormwood *(A. afra)* is a perennial woody shrub with a rich, notable historical background in traditional medicine. It is used to treat a variety of diseases, including cough, fever, sore throat, asthma, TB, internal worm infestation, cold, pneumonia, and diabetes, among others (Kane et al. 2019). In 2020, it gained significant attention after traditional medicinal practitioners claimed its potential in treating COVID‐19 symptoms (Binyane 2022), and subsequently, research highlighted its potential role in treating respiratory diseases and COVID‐19 symptoms (Adeleye et al. 2021). As a result, local communities have been harvesting *Artemisia afra* unsustainably to address COVID‐19 symptoms, leading to more concerns over its ecological impact and long‐term availability.

Despite its wide natural abundance and distribution, as well as its medicinal properties, *A. afra* faces alarming unsustainable harvesting practices (Guisan and Thuiller 2005; West et al. 2016). These plants thrive in specific climates and topo‐environmental conditions, particularly found in mountainous areas with altitudes ranging from 1500 to 3000 m (Patil et al. 2011), and subtropical areas characterised by an average yearly precipitation of above 650 mm (Setshedi et al. 2022). It prefers growing in well‐drained soil types such as sandy loam, volcanic ash, granitic sands and loam, characterised by a pH of between 5.0 and 7.5 (Braünlich et al. 2018; Quattrocchi 2012). For instance, the Limpopo Province in South Africa provides a suitable environment with an average yearly precipitation of 1500 m and an average summer temperature of 23°C (Hersbach et al. 2020).

In response to the unsustainable harvesting practices of *A. afra* , there is a vital need to predict its suitable habitats on the basis of the favourable climate and topo‐environmental variables using geospatial technologies. Remote sensing and Geographical Information Systems (GIS), along with the integration of robust machine learning algorithms, have revolutionised the capability to rapidly and accurately predict the distribution of medicinal plants over a larger geographic region (Shamsudeen 2020; Cui et al. 2023). Various machine learning algorithms, such as Maximum entropy (MaxEnt), K‐nearest neighbor (K‐NN), decision tree (DT), fuzzy rule‐based systems (FRBS), adaptive neuro‐fuzzy inference system (ANFIS), deep neural networks (DNN), ANN, support vector machine (SVM), and random forest (RF), are the most widely used to analyse species distribution trends where location and environmental data are scarce in order to support management and conservation efforts (Mashao et al. 2023).

The ongoing issue of the unsustainable harvesting of *A. afra* has raised concerns about the efficient management and conservation of these valuable medicinal plants. Gaining a comprehensive understanding of the distribution patterns and the key climatic and topo‐environmental conditions influencing the spread of these species is crucial for their effective management and conservation efforts. This knowledge will lead to the development of effective strategies for sustainable harvesting, the conservation of critical habitats, and the long‐term survival of *A. afra* and its associated ecosystems. Active engagement of communities and stakeholders is crucial for promoting sustainable harvesting and biodiversity conservation in areas where this plant is found. To address these challenges, this study utilized MaxEnt, a machine learning approach to predict suitable habitats for *A. afra* across the Greater Sekhukhune District municipality. This paper aims to support the sustainable management and conservation of this important medicinal plant.

## 2 Materials and Methods

### 2.1 Study Area

**FIGURE 1:**
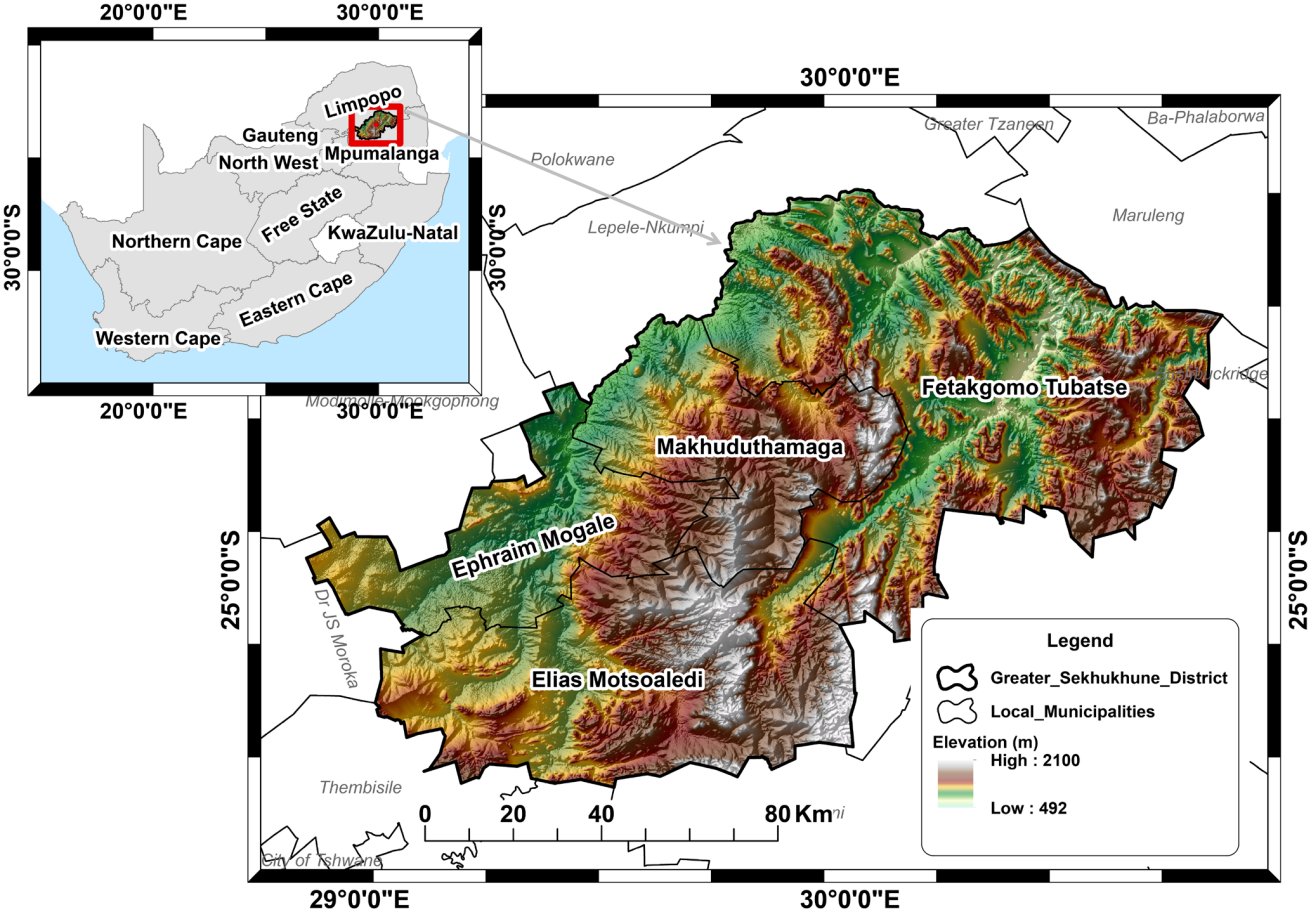
Location of the South African and Sekhukhune District Municipality.

The study was conducted in the Greater Sekhukhune District Municipality (GSDM), located in the Limpopo Province of South Africa, as depicted in Figure 1. The GSDM is geographically situated within 24° 51′ 19″ S and 29°58′47″ E coordinates, covering a spatial extent of approximately 13,528 km^2^ (13,528,000 ha). This district is home to a population of approximately 1,336,805 individuals, primarily comprising communal farms and rural settlements (StatsSA (Statistics South Africa) 2022).

The communal farmlands dominate 70% of the area, whereas commercial land uses cover the remaining 30% (Sepuru and Dube 2018). The climate in the GSDM exhibits distinct seasonal variations, with an average summer temperature of approximately 23°C and a winter temperature that may drop to around 19°C (Hersbach et al. 2020). The district also experiences an average annual rainfall of about 560 mm (Mpandeli et al. 2015; Hersbach et al. 2020). The geography of the GSDM is characterised by hilly to mountainous terrains, with an altitude ranging from 490 to 2101 m above sea level.

### 2.2 *Artemisia afra* Species Distribution Data

Field surveys were conducted from 1st November to 10th November 2021, within the geographical boundaries of the GSDM. Datasets were collected during the field survey using the Garmin GPS devices to accurately record the XY coordinates of the exact locations of *A. afra* and the adjacent land cover types. A total of 105 sampling points were successfully collected across the sampled local municipalities with the SGDM. In our study, we employed a data split approach, where 70% of the sampled points were used for training and the remaining 30% were used for testing the MaxEnt model. This approach was employed to ensure the model's robustness and accuracy by training it on a substantial portion of the available data and then validating it on a separate set of data points (He et al. 2023).

### 2.3 Environmental Variable for Assessing the Distribution of *Artemisia afra*

This study defined suitable habitats for *A. afra* as a potential area to be dominated by the distribution of *A. afra* on the basis of climate and topo‐environmental conditions. Because of the necessity for careful selection of relevant environmental variables to produce reliable predictions 105 sampled XY coordinates datasets gathered during a field survey and 27 candidate environmental variables obtained and derived from various open and free sources (Table 1) were utilised to develop a suitable habitat map for *A. afra* using the MaxEnt model. In this study, environmental variables were selected on the basis of ecological relevance and data accessibility. Correlation analysis was initially conducted using 27 possible environmental variables (19 bioclimatic and 8 topo‐environmental) to avoid multicollinearity issues (Liu et al. 2022).

**TABLE 1:** Climate and topo‐environmental variables used in developing the MaxEnt model.

Environmental variables	Units	Resolution	Source
Bio1: Annual mean temperature	°C	30 arc s	WorldClim (https://www.worldclim.org/)
Bio2: Mean diurnal range	°C	30 arc s	WorldClim (https://www.worldclim.org/)
**Bio3: Isothermally (BIO2/BIO7) (× 100)**	%	30 arc s	WorldClim (https://www.worldclim.org/)
Bio4: Temperature seasonality (standard deviation ×100)	%	30 arc s	WorldClim (https://www.worldclim.org/)
Bio5: Maximum temperature of warmest month	°C	30 arc s	WorldClim (https://www.worldclim.org/)
Bio6: Minimum temperature of coldest month	°C	30 arc s	WorldClim (https://www.worldclim.org/)
Bio7: Temperature annual range (Bio5‐Bio6)	°C	30 arc s	WorldClim (https://www.worldclim.org/)
Bio8: Mean temperature of wettest quarter	°C	30 arc s	WorldClim (https://www.worldclim.org/)
Bio9: Mean temperature of driest quarter	°C	30 arc s	WorldClim (https://www.worldclim.org/)
**Bio10: Mean temperature of warmest quarter**	°C	30 arc s	WorldClim (https://www.worldclim.org/)
Bio11: Mean temperature of coldest quarter	°C	30 arc s	WorldClim (https://www.worldclim.org/)
Bio12: Annual precipitation	mm	30 arc s	WorldClim (https://www.worldclim.org/)
Bio13: Precipitation of wettest period	mm	30 arc s	WorldClim (https://www.worldclim.org/)
**Bio14: Precipitation of the driest period**	mm	30 arc s	WorldClim (https://www.worldclim.org/)
**Bio15: Precipitation seasonality (cv)**	%	30 arc s	WorldClim (https://www.worldclim.org/)
Bio16: Precipitation of wettest quarter	mm	30 arc s	WorldClim (https://www.worldclim.org/)
Bio17: Precipitation of driest quarter	mm	30 arc s	WorldClim (https://www.worldclim.org/)
**Bio18: Precipitation of the warmest quarter**	mm	30 arc s	WorldClim (https://www.worldclim.org/)
Bio19: Precipitation of coldest quarter	mm	30 arc s	WorldClim (https://www.worldclim.org/)
**Elevation**	Degree	30 m	SRTM DEM: (https://code.earthengine.google.com/)
**Slope**	Degree	30 m	Retrieved from DEM
**Aspect**	Degree	30 m	Retrieved from DEM
**Topographic wetness index**	Degree	30 m	Retrieved from DEM
**Soil**	—	1:1 million	SOTER Database (https://www.isric.org/explore/soter)
**Land cover**	—	30 m	https://egis.environment.gov.za/gis_data_downloads)
**Normalised difference vegetation index**	—	30 m	Sentinel‐2 MSI (https://code.earthengine.google.com/)
**Distance from rivers**	m	1:50,000	Rivers (https://www.dws.gov.za/iwqs/gis_data/river/)

*Note:* Bold text indicates extracted environmental variables for model construction after screening.

Abbreviation: CV, coefficient of variation.

Highly correlated variables (*r* > 0.7) were assessed on the basis of significance and expert opinion from the preliminary SDMs testing. Finally, only 13 variables were retained, including 5 bioclimatic and 8 topo‐environmental variables (Table 1). The adequacy of the number of sampled XY points was determined on the basis of the proven performance of the MaxEnt modeling technique, which has consistently produced accurate results with a minimum of 10 sample points (Mashao et al. 2023). This study exclusively focused on areas where *A. afra* was located within the district, as the MaxEnt model requires the use of present‐only data (Bartoňová 2019).

The climatic and topo‐environmental variables used to develop *A. afra* suitable habitat maps in this study were chosen on the basis of literature and their relevance in modelling suitable habitats for the distribution of *A. afra* as well as supported by many previous studies (Hosseini et al. 2013; Esfanjani et al. 2018; Zhang et al. 2019; Khan et al. 2022; Shao et al. 2022). The chosen bioclimatic variables included: isothermally (Bio3), mean temperature of the warmest quarter (Bio10), precipitation of the driest period (Bio14), precipitation seasonality (Bio15), and precipitation of the warmest quarter (Bio18) (Table 1). For topographic variables (Table 1), elevation, slope, aspect, and topographic wetness index (TWI) were selected because they are crucial for the growth and persistence of *A. afra* . Furthermore, environmental predictors were considered, including land cover, soil types, normalized difference vegetation index (NDVI), and proximity to rivers (Table 1), as they influence hydrology and vegetation dynamics. Before incorporating the variables into the MaxEnt model, a series of crucial pre‐processing steps were undertaken to standardize the climatic and topo‐environmental data. These steps were instrumental in ensuring that all variables were uniform and compatible (Mashao et al. 2023; He et al. 2023).

Firstly, all vector variables were converted into raster format, and subsequently, all variables were projected to World Geodetic System 84, Universal Transverse Mercator Zone 35 South (WGS_1984_UTM_Zone_35S). Secondly, variables were resampled to 30 m by 30 m spatial resolution, and variables were masked using the variable with the smallest values in both columns and rows. Finally, all variables were converted into the format (ESRI ASCII), which is supported by MaxEnt using ArcGIS 10.8 software (He et al. 2023; Mashao et al. 2023). These steps were crucial to produce the specific data format required by the MaxEnt software (He et al. 2023).

### 2.4 Maximum Entropy Model (MaxEnt)

The MaxEnt software (https://biodiversityinformatics.amnh.org/open_source/maxent/ accessed on 12 June 2022) is a widely adopted tool in ecological and biogeographical studies for predicting species distributions (Hosseini et al. 2013; Mashao et al. 2023). This model employs machine learning techniques to establish correlations between species occurrence data and environmental variables, thereby determining the likelihood of a species' presence at each location (Hosseini et al. 2013; Abolmaali et al. 2018). To avoid overfitting, which can happen when the model becomes overly complex and fits the noise in the data rather than the true relationships, a regularization multiplier is used (Chahouki and Sahragard 2016; Dai et al. 2022). For this study, the logistic output format was selected, enabling the prediction of regions with high suitability for the species.

The jack‐knife test, a commonly used procedure, helps determine the significance of each predictor variable in identifying a species' distribution. It involves systematically removing individual variables and rerunning the model to see how its performance changes (Shcheglovitova and Anderson 2013; Mashao et al. 2023; He et al. 2023). This process aids in identifying the most influential variables in predicting species distributions. When there are no distribution probability assumptions, our research uses the gain of environmental variables to evaluate the significance of each predictor variable (He et al. 2023).

### 2.5 Model Evaluation

The accuracy of the MaxEnt prediction model was evaluated by generating a Receiver Operating Curve (ROC) for the test data and calculating the Area Under the Curve (AUC) (Phillips 2005; Wang et al. 2024; Çoban et al. 2020; Mashao et al. 2023). The AUC, a widely used statistic for measuring the accuracy of species distribution models, can range from 0 to 1. A model performs better if the AUC is closer to 1, whereas an AUC closer to 0.5 indicates performance no better than random prediction (Phillips and Elith 2010; Çoban et al. 2020; Mashao et al. 2023). An AUC between 0.7 and 0.9 signifies acceptable model performance, whereas an AUC greater than 0.9 indicates good performance (Zhao et al. 2018; He et al. 2023).

In this study, the AUC was used to evaluate the MaxEnt model's ability to predict the conditions conducive to the growth of *A. afra* . The threshold values for interpreting the AUC values were on the basis of established guidelines (Çoban et al. 2020; He et al. 2023). AUC values less than 0.8 are considered poor, those between 0.8 and 0.9 are deemed acceptable, and values above 0.9 are considered excellent (Çoban et al. 2020; Mashao et al. 2023). The AUC values were further classified into five groups to represent the predicted habitat suitability for *A. afra* , ranging from none (0–0.07) to very high (0.75–1). The suitable habitat was further divided into areas of non‐suitable, low suitability (0.8–1.9), medium suitability (1.9–3.4), high suitability (> 3.4–5.2), and very high suitability (> 5.2) using natural breaks.

## 3 Results

### 3.1 Performance of the Model in Predicting a Suitable Habitat for *Artemisia afra*

**FIGURE 2:**
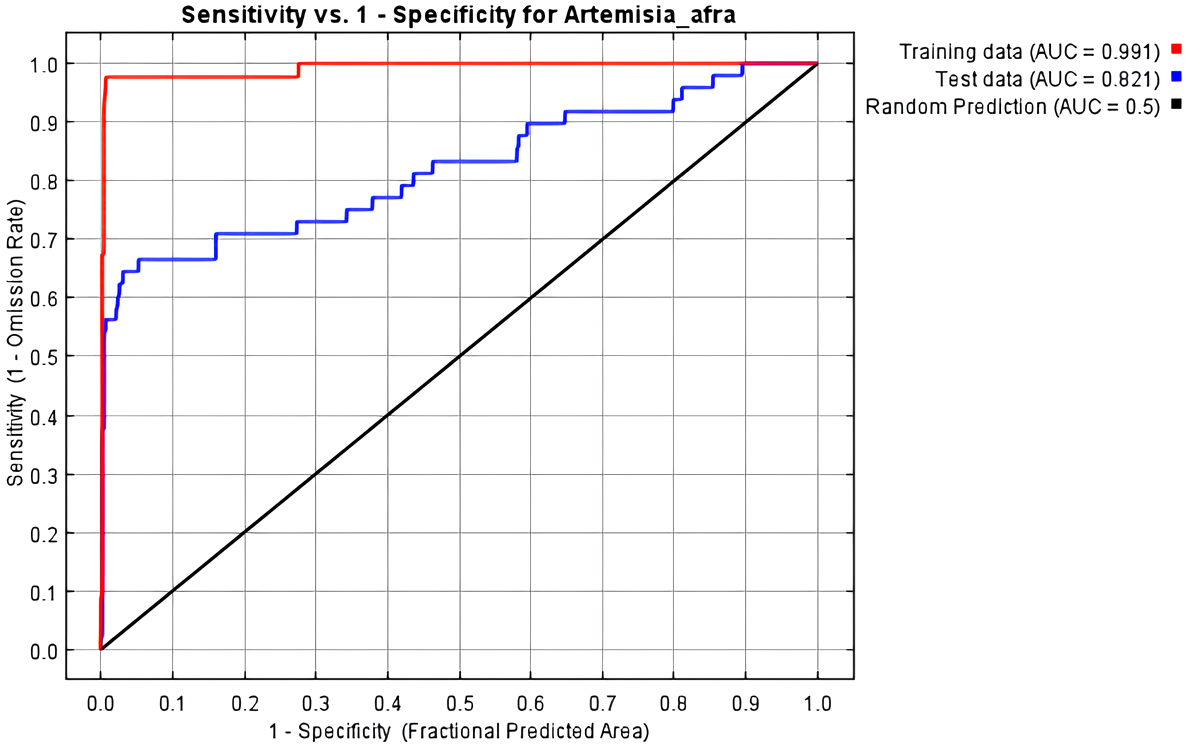
The receiver operating characteristic (ROC) curve, with the training data fit represented by a red line and the testing data fit by a blue line. The expected line for a random model is depicted in black. This visual representation allows for a clear comparison between the model's performance and a random prediction.

**FIGURE 3:**
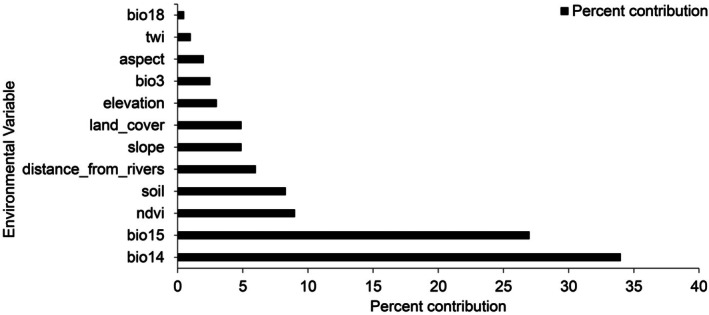
The percentage contribution of each environmental variable in predicting suitable habitats for *Artemisia afra* within the Sekhukhune district municipal area. This visualisation provides a clear understanding of the relative importance of each variable in determining the species' habitat suitability.

The AUC for both the training and testing data, along with the sensitivity and specificity, indicates a high level of model accuracy in predicting suitable habitats for *A. afra* (Figure 2). The model demonstrated good performance compared to random prediction, with an average AUC of 0.91, indicating excellent results from both the training and testing datasets. Five factors contributed to over 80% of the variance in the model: soil, distance from rivers, NDVI, Bio14 and Bio15. Among these, Bio14 had the most significant influence on predicting the distribution of *A. afra* , accounting for 33.6% of the variation, as shown in Figure 3. This highlights the significance of these environmental variables in determining suitable habitats for *A. afra* .

In addition to utilising 25% of the sample data for internal testing (AUC = 0.82), the predicted habitat suitability map underwent visual and ecological cross‐validation against identified *A. afra* distributions in the Sekhukhune district, incorporating regions identified through field surveys and traditional knowledge sources. This method aligns with validation techniques employed in comparable research (Tesfamariam et al. 2022; Wang et al. 2024), wherein model results were assessed against ecological ground‐truthing or expert‐identified biodiversity hotspots. The spatial alignment of high‐suitability areas with identified ecological habitats increases confidence in the model's predictive strength.

### 3.2 Response Analysis of the Main Environmental Variables Affecting the Distribution of *Artemisia afra*

**FIGURE 4:**
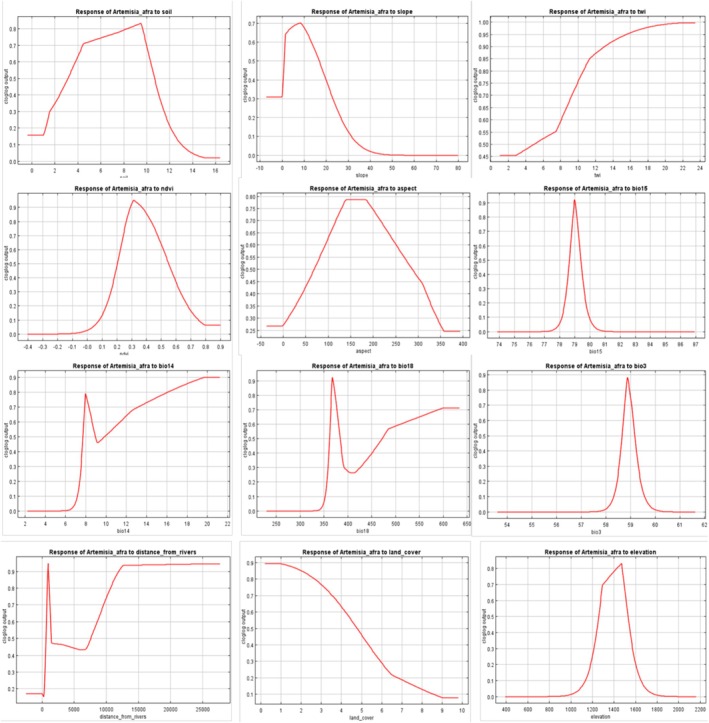
The influence of key environmental factors on the distribution of *Artemisia afra* in the Greater Sekhukhune District municipality. It specifically highlights the impact of altitude, mean annual precipitation, and the average temperature of the warmest month on the distribution of *Artemisia afra* under varying environmental conditions.

A strong correlation was observed between the distribution of *A. afra* in the Greater Sekhukhune district municipality and the dominant environmental variables. Figure 4 illustrates the response curve between these dominant environmental variables and the distribution probability derived from the MaxEnt model. This curve reflects the range of environmental variable values for different thresholds. The maximum sum of the specificity and sensitivity of the training data was 0.32, which served as the threshold for classifying the habitat into non‐suitable and suitable zones.

The analysis found key factors influencing the distribution of *A. afra* in the Greater Sekhukhune District Municipality. Optimal conditions include soil thicknesses of 1.9–15.5 m, slopes from 0.35° to 30.5°, and NDVI values between 0.1% and 0.8%. Other factors included a TWI ranging from 7.8° to 23.7°, an aspect from 0.1° to 350.2°, and precipitation levels (Bio15, Bio14, Bio18) within specified ranges. The distance from rivers ranges from 5040 to 25,000 m, land cover varies from 0.2% to 9%, and altitudes extend from 1000 to 1700 m. The above minimum and maximum values combined indicate the probability of the species occurrence in the study region.

### 3.3 Prediction Map

**FIGURE 5:**
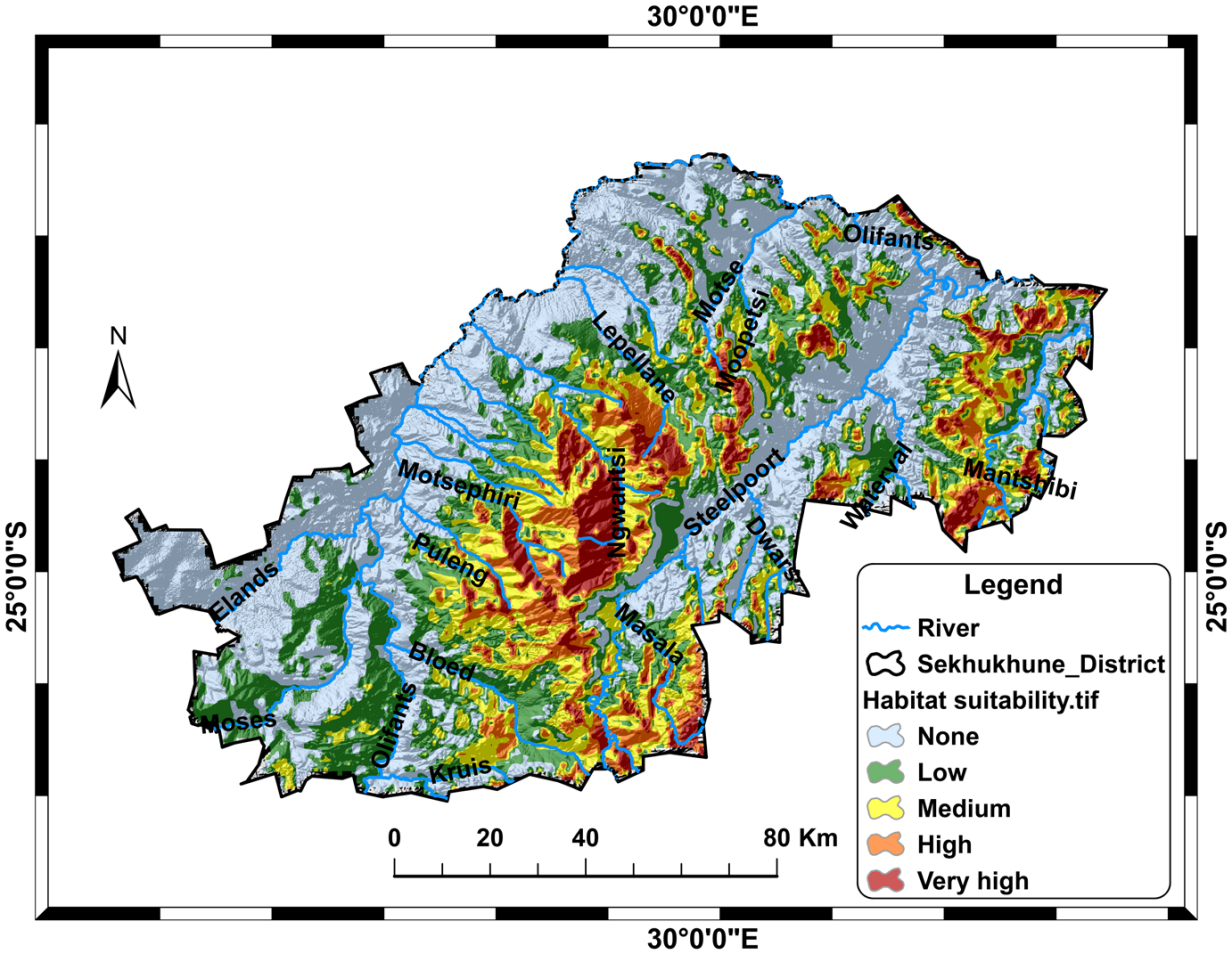
Presents the distribution of suitable habitats for *Artemisia afra* within the Sekhukhune district.

The MaxEnt model was employed with 15 environmental variables to identify potential habitats for *A. afra* . The output of the MaxEnt model was reclassified using the Reclass tool in ArcGIS v10.7 software. The natural‐breaks classification method (Jenks) was employed to classify the predicted suitable area of 674.79 ha into five different suitability levels: very high suitability (69.0 ha), high suitability (106.20 ha), medium suitability (185.56 ha), and low suitability (313.98 ha), as shown in Table 2. Flat regions, covering an area of 564.07 ha, are classified as non‐suitable (Table 2). The results also revealed that areas with low to medium suitability are primarily found in low‐lying regions, with occasional distribution in mountainous areas (Figure 5). The distribution of *A. afra* is closely associated with rivers. Areas with very high to high suitability are primarily located in mountainous regions and along riverbanks.

**TABLE 2:** The index values for habitat suitability, the corresponding area coverage, and associated colour codes used in Figure 5: light blue = None (0–0.8), green = Low (0.8–1.9), yellow = Medium (1.9–3.4), orange = High (3.4–5.2), red = Very high (5.2–9.7).

Habitat suitability	Index	Area (sqkm)	Area (%)
None	0–0.8	564.07	45.53
Low	0.8–1.9	313.98	25.34
Medium	1.9–3.4	185.56	14.98
High	3.4–5.2	106.20	8.57
Very high	5.2–9.7	69.05	5.57

## 4 Discussion

Concerns persist about the drastically increased rates of habitat loss for species resulting from climate change (Ngarega et al. 2024). However, considerable research remains to be conducted on the relationships between species vulnerability and environmental variables. Furthermore, a species' habitat is essential for population growth, reproduction, and survival, and changes in its quality can have a direct impact on the distribution and abundance of the species (Zhu et al. 2020; Ngarega et al. 2024). To the best of our knowledge, this is one of the first studies to employ distributional modelling to forecast *A. afra's* potential distribution in South Africa under the current changing climate (Mofokeng et al. 2024). In light of the current shifting climate, this work, to our knowledge, is the first to use distributional modelling to predict the potential distribution of *A. afra* in South Africa.

The advancement in geospatial technologies and machine learning models for predicting suitable habitats has emerged as an effective tool for evaluating habitat suitability and conserving natural resources, particularly for the conservation of medicinal and aromatic plants. This study focused on predicting suitable habitats for *A. afra* in the Sekhukhune District Municipality using the MaxEnt model. The MaxEnt analysis results indicated that the spatial distribution of *A. afra* is influenced by several variables, including soil type, proximity to rivers, NDVI, and precipitation (Bio14 and Bio15). Precipitation emerged as a critical variable in predicting suitable habitat distributions for *A. afra* . The study found that areas with precipitation levels below 1200 mm and elevations above 1295 m above sea level are predicted to be unsuitable habitats.

The performance of the MaxEnt model was evaluated to enhance the accuracy of the predicted suitable habitats for *A. afra* . The model's performance was assessed by examining its stability and accuracy in making predictions. This was achieved by using the ROC curve to evaluate the model's stability and the AUC values to measure its prediction accuracy. The findings revealed that the MaxEnt model performed exceptionally well in predicting the suitable habitats for *A. afra* , as substantiated by the average AUC value of 0.91. Our findings on *A. afra's* potential distribution prediction were consistent with those of Ngarega et al. (2024), who demonstrated that the MaxEnt model performed exceptionally well, considering the average AUC of 0.953.

According to the current potential distribution findings, *A. afra* 's potential distribution areas are distributed in the eastern, southern, and central regions of the district. The recorded location of this species showed a significant correlation with its predicted potential range. Additionally, the results of this study will offer locations for field surveys and studies on the persistence and distribution of *A. afra* . It is well known that *A. afra* grows on plains, bottom slopes, and riverbank edges; these results are consistent with those of Setshedi et al. (2022), who reported that *A. afra* is dominant in the bottom slopes and plains of the Klipriviersberg Nature Reserve.

The model predicted a total suitable area of 674.79 ha, with high to very high suitability areas primarily found in mountainous regions and near rivers. Conversely, low to medium‐suitability areas were predominantly located in low‐lying areas, with occasional sightings in mountainous regions. The species distribution closely followed riverbanks, indicating the significance of proximity to rivers in predicting suitable habitats for *A. afra* . These findings can be leveraged to devise conservation strategies for *A. afra* and predict potential changes in the species' distribution because of variations in environmental variables.

The results align with those of Yi et al. (2016), who identified seven key variables that significantly impact determining the appropriate habitat for *Homonoia riparia* Lour. These variables included elevation, precipitation seasonality, precipitation of the coldest quarter, distance to the nearest river, temperature seasonality, precipitation of the driest month, and annual mean temperature. The use of the MaxEnt model proved to be an effective method for predicting suitable habitats for medicinal plants, such as *A. afra* . This model can be applied in other regions to develop conservation strategies for endangered plant species. The study, conducted by Yang et al. (2013), employed MaxEnt modeling to investigate the distribution of the Malabar nut medicinal plant in the Lesser Himalayan foothills.

The model identified key environmental factors affecting the species' habitat suitability, including elevation, precipitation seasonality, distance to the nearest river, temperature seasonality, and annual mean temperature. The model demonstrated high accuracy, with an AUC value of 92.3, making it a promising tool for predicting potential distribution and aiding species restoration and conservation efforts. Lastly, a variety of socioeconomic factors, including anthropogenic disturbance, land‐use/cover change, edaphic factors, climate, and topographic variables, among others, may impact a species' distribution. Under some conditions, because of the influence of microclimates, regions that are predicted to be low to medium suitability are *A. afra* 's potential distribution range. Therefore, the results of our study are only indicative and should be interpreted with caution. The modeling approach employed in this study is applicable to other regions with similar ecological contexts, facilitating wider spatial assessment of *A. afra* distribution.

This study included 27 environmental variables, which were adjusted to include 13 ecologically relevant predictors. However, it is evident that certain fine‐scale environmental or anthropogenic factors, such as specific soil micro‐properties, land‐use history, and localized human disturbances, were excluded because of data limitations. The robust model performance (AUC = 0.91) demonstrates that the selected variables effectively represent the primary environmental variations that influence the distribution of *A. afra* in the region. Future research needs to incorporate these additional variables, such as high‐resolution data and human influence, to improve the model and increase its relevance for site‐specific conservation planning.

## 5 Conclusion

The model's performance in predicting suitable habitats for *A. afra* in the Greater Sekhukhune district municipality was found to be excellent, with an average AUC of 0.91. The model's superior performance over random prediction is a testament to its robustness and reliability. The study identified five key environmental variables—soil, distance from rivers, NDVI, Bio14, and Bio15—that accounted for over 80% of the variance in the model. Among these, Bio14 had the most significant impact, accounting for 33.6% of the variation. This highlights the importance of these environmental variables in determining the suitable habitats for *A. afra* . The response analysis of the main environmental variables affecting the distribution of *A. afra* revealed a strong correlation between the species' distribution and these variables.

The optimal ranges of these variables were identified, providing valuable insights into the environmental conditions favourable for the distribution of *A. afra* . The MaxEnt model predicted a total suitable area of 674.79 ha for *A. afra* , divided into different suitability levels. The results revealed that areas with low to medium suitability are primarily found in low‐lying regions, with occasional distribution in mountainous areas. This study provides a comprehensive understanding of the environmental conditions and variables influencing the distribution of *A. afra* . It also highlights the model's effectiveness in predicting suitable habitats for the species, thereby contributing to conservation efforts and the sustainable management of *A. afra* in the Greater Sekhukhune district municipality. Furthermore, to achieve more realistic conservation outcomes, future modelling initiatives should integrate human‐related factors, such as land‐use change, habitat fragmentation, and harvesting pressures, which may substantially impact the distribution patterns of *A. afra* , extending beyond the limits of mainly environmental predictors.

### 5.1 Conservation Recommendations

Integrating local communities into management strategies is crucial for the long‐term conservation of *A afra*. We recommend establishing participatory conservation initiatives that engage traditional healers, community leaders, and local environmental forums. These initiatives could encompass awareness campaigns, collaboratively designed restoration projects, and community‐driven monitoring of essential habitats. The outputs from this study can be utilized to pinpoint priority areas for collaborative conservation efforts. Working together with local schools, health practitioners, and agricultural cooperatives will significantly improve the sharing of knowledge and the sustainability of conservation measures.

## Author Contributions

**Willem Matsane:** conceptualization (lead), data curation (lead), formal analysis (lead), investigation (lead), methodology (lead), project administration (lead), resources (lead), software (lead), validation (lead), visualization (lead), writing – original draft (lead), writing – review and editing (lead). **Inos Dhau:** supervision (equal). **Mologadi Clodean Mothapo:** conceptualization (equal), methodology (supporting), project administration (supporting), software (supporting), supervision (supporting), validation (supporting), visualization (supporting), writing – review and editing (supporting). **Kgabo Humphrey Thamaga:** writing – review, and editing (supporting).

## Conflicts of Interest

The authors declare no conflicts of interest.

## Data Availability

The data supporting the findings of this study are accessible in an open‐access repository at: https://doi.org/10.5061/dryad.dz08kps71.
